# Hydrophobic Mesoporous Silica-Coated Solid-Phase Microextraction Arrow System for the Determination of Six Biogenic Amines in Pork and Fish

**DOI:** 10.3390/foods12030578

**Published:** 2023-01-28

**Authors:** Mengfei Chen, Hangzhen Lan, Daodong Pan, Tao Zhang

**Affiliations:** State Key Laboratory for Quality and Safety of Agro-products, Key Laboratory of Animal Protein Deep Processing Technology of Zhejiang Province, Zhejiang-Malaysia Joint Research Laboratory for Agricultural Product Processing and Nutrition and College of Food and Pharmaceutical Sciences, Ningbo University, Ningbo 315800, China

**Keywords:** functionalized mesoporous silica, solid-phase microextraction arrow, biogenic amines, pork, fish

## Abstract

In this study, a functionalized mesoporous silica-coated solid-phase microextraction (SPME) Arrow system was developed for the enrichment of six biogenic amines (BAs) from pork and fish samples before gas chromatographic separation with a mass spectrometer as a detector. MCM-41 was utilized as the substrate material and thereby functionalized by titanate and sodium dodecyl sulfate to adjust its surface acidity and hydrophobicity, respectively. The functionalized MCM-41 (named as MCM-T-H) was coated on a bare SPME Arrow using the dipping method and polyacrylonitrile was used as the adhesive. The extraction capacity and selectivity of the MCM-T-H-SPME Arrow for six kinds of derivatized BAs were studied and compared with commercial SPME Arrows. Experimental parameters, e.g., sample volume, derivatization reagent amount, extraction time, and desorption time, which have a dramatic effect on SPME Arrow pretreatment, were optimized. Acidity enhanced MCM-T-H coating showed a much higher affinity to derivatized BAs compared to a commercial SPME Arrow in terms of extraction capacity. In addition, hydrophobicity modification significantly reduced the interference of water molecules on the interaction between MCM-T-H and the derivatized BAs. The MCM-T-H-SPME Arrow showed efficient separation and enrichment capacity for derivatized BAs from complex matrices and therefore, the sample pretreatment time was saved. According to the experimental results, the optimal condition was to add 10 μL derivatization reagent to a 10 mL sample and maintain an agitation speed of 1250 r min^−1^. The MCM-T-H-SPME showed excellent reproducibility (RSD < 9.8%) and fast adsorption kinetics (30 min) and desorption kinetics (5 min) for derivatized BAs under optimal conditions. In summary, the MCM-T-H-SPME Arrow based method was employed for accurate monitoring of the variations of BAs in pork and fish, and good results were achieved.

## 1. Introduction

Biogenic amines (BAs) are nitrogen-containing compounds with low-molecular weight. Their chemical structures contain one or more amino groups (NH_2_) which show biological activity in living beings [1]. BAs are generated by the bacterial decarboxylation of amino acids where the α-carboxyl groups are removed from precursor amino acids [2]. BAs are naturally present in meat, seafood, fish, and wine and they are widely regarded as food spoilage markers because higher concentrations of BAs can be detected when the food spoilage is worse [3]. Tryptamine (TRY), phenylethylamine (PHE), putrescine (PUT), cadaverine (CAD), histamine (HIS), tyramine (TYR), spermidine (SPD), and spermine (SPM) are the most commonly generated BAs during food spoilage [4,5]. Furthermore, an excessive intake of BAs from spoiled food can not only cause adverse effects on the human nervous and respiratory system but can also lead to anaphylactic shock and death [5,6]. Therefore, monitoring BA content in foods is very important to guarantee food quality and safety. However, detecting BAs with wide concentration ranges in complex food matrices is not a straightforward task. It is essential to establish sensitive, wide-linear range, and selective analytical methods for the detection of BAs.

Sample pretreatment is the key step before instrumental detection. It is normally tedious and time consuming, and any inaccuracies in the process can confuse the analytical results [7]. Traditional detection methods of BAs require complex sample pretreatment, including homogenization, extraction, purification, and derivatization, and consume a lot of organic solvents, which greatly limits the rapid detection of BAs. Pretreatment technique is essential prior to the detection of BAs. Liquid–liquid extraction [8,9,10], dispersed liquid–liquid microextraction [11,12], dispersed solid-phase extraction [13], solid-phase microextraction (SPME) [14], and other sample preparation methods are commonly used for sample pretreatment for the detection of BAs. Among them, SPME gained more and more attention owing to its merits of combination sampling, separation, enrichment, and injection into one step, which simplified the operational steps, saving reagent consumption and improving the efficiency of analysis [15]. In recent years, a new prototype, the SPME Arrow method, has been developed [16]. The SPME Arrow overcomes drawbacks associated with traditional SPME fiber, such as small extraction phase volumes, poor interdevice reproducibility, and limited mechanical robustness [17]. In order to achieve the separation of BAs, the adsorbent hosted on an SPME Arrow needs to be customed.

MCM-41 is a type of mesoporous silicon material [18], which has the characteristics of a stable framework, a high specific surface area (700–1500 m^2^ g^−1^), and a large pore volume (0.5–3.0 cm^3^ g^−1^). The aperture distribution is narrow, and the material with a diameter of 20–300 Å can be made by changing the manufacturing process [19]. However, the most promising characteristic of MCM-41 is its natural acidic surface that predicts its high affinity to basic amines. In our previous study [20], acidified MCM-41 groups showed an exceptional affinity to basic volatile aliphatic amines. Therefore, MCM-41 was selected as the adsorbent to separate basic BAs from food samples. Some studies have shown that its acidic characteristics can be improved when MCM-41 contains Ti [21]. On the other hand, BAs are polar and nonvolatile. Therefore, in the process of sample pretreatment, it is necessary to derivatize the BAs which are dissolved in water. In this process, water molecules have significant interference with the extraction of derivatized BAs through hydrophilic adsorbents. Therefore, it is necessary to improve the hydrophobicity of MCM-41.

**Figure 1:**
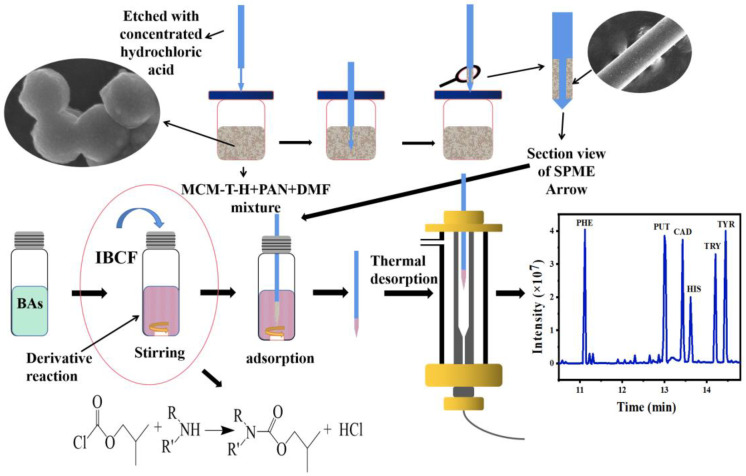
Schematic illustration of an SPME Arrow GC–MS method for biogenic amines detection.

Sodium dodecyl sulfate (SDS) is a kind of surfactant that is soluble in water and its ester linkage can be hydrolyzed when heated. This creates a hydrophobic surface for the particles and improves the compatibility between the particles and the analytes [22]. Nikosokhan et al., used SDS to fabricate hierarchical cobalt-based superhydrophobic coating with nano- and microscale roughness. The results showed that the optimal superhydrophobic surface can be prepared by using SDS [23]. Therefore, MCM-41 was functionalized by titanate (TTIP) and SDS to obtain acidic and hydrophobic MCM-T-H. The synthesis route of the materials is shown in Figure S1. MCM-T-H was mixed with polyacrylonitrile (PAN), and then coated on an SPME Arrow by a simple dipping approach (Figure 1). This method can make the material uniformly adhere to the surface of the metal rod, prevent the aggregation of MCM-T-H particles, and provide more active adsorption sites. This method inherits the advantages of high specific surface area and high-adsorption capacity of MCM-T-H. It can also improve the mechanical properties and antifouling ability of the material and prolong the usage of the SPME Arrow.

There are many classic analytical methods for the determination of BAs such as thin-layer chromatography [24], ion chromatography [25], capillary electrophoresis [26], high-performance liquid chromatography [27,28] and high-performance liquid chromatography with tandem mass spectrometric [29]. In addition, gas chromatography–mass spectrometry (GC–MS) is also a common analytical method, which has the advantages of good robustness, simple operation, and low cost. The derivatized BAs are volatile and are easily decomposed during heating, which make them possible to be detected by GC–MS.

The purpose of this study was to prepare the functionalized mesoporous silica MCM-T-H with excellent adsorption capacity and fast kinetic extraction to derivatized BAs from different sample matrices. MCM-T-H was synthesized based on mesoporous MCM-41 and was then utilized as the coating material of the SPME Arrow using a straightforward dipping strategy. MCM-T-H series materials were fully characterized to investigate the extraction mechanism of MCM-T-H to derivatized BAs. The preparation parameters that affected the MCM-T-H coating extraction performance were also studied. After optimizing the SPME parameters, the applicability of MCM-T-H SPME Arrows was further evaluated by exploiting them for the detection of BAs in pork and mackerel samples.

## 2. Materials and Methods

### 2.1. Reagents

Phenylethylamine (PHE), putrescine (PUT), cadaverine (CAD), histamine (HIS), tryptamine (TRY), tyramine (TYR), cetyltrimethylammonium bromide (CTAB), TTIP, SDS, tetraethyl silicate (TEOS), ethanol, polyacrylonitrile (PAN), isobutyl chloroformate (IBCF), N-N dimethylformamide (DMF), trichloroacetic acid (TCA), sodium hydroxide (NaOH), and ammonium hydroxide solution (NH_4_OH) were purchased from Macklin (Shanghai, China). Anhydrous toluene and hydrochloric acid (HCl) were from Luoyang Haohua Chemical Reagent Co., Ltd. (Henan, China). The ultrapure water used in the study was from a ultrapure water system (Chengdu Yinghang Water Treatment Equipment Co., Ltd.) with a resistivity of 18.25 MΩ·cm. The headspace bottle and PTFE/silicone septum screw cap were from Merck KGaA (Shanghai, China). The commercial SPME Arrow with the Divinylbenzene/Carboxen/Polydimethylsiloxane (DVB/CAR/PDMS) coating and the bare SPME Arrow were purchased from CTC Analytics (Zwingen, Switzerland). The stock solutions containing six BAs were prepared by dissolving them in HCl at 0.1 mol L^−1^ and stored at 4 °C. A series of working solutions were obtained by diluting the stock solutions properly with ultrapure water. Then the pH of the working solutions was adjusted to 11 with 2 mol L^−1^ NaOH. For each SPME Arrow extraction, 10 mL of the working solution was added to the sample vial for the subsequent SPME Arrow extraction.

### 2.2. Instruments

The detection of BAs was conducted by gas chromatography–mass spectrometry (GC–MS) (Agilent 8890-5977B, Agilent Technologies Inc., Palo Alto, CA, USA). The chromatographic column was a HP-5 MS (30 m × 250 μm × 0.25 μm, Agilent Technologies Inc., Palo Alto, CA, USA). Surface groups of the materials were analyzed by a fourier transform infrared spectrometer (FTIR) (Nicolet 6700, Thermo Fisher Scientific, Madison, WI, USA). Powder X-ray diffraction (XRD) patterns were recorded on a Bruker D8 Advance (Bruker Co. Ltd., Karlsruhe, Germany). Transmission electron microscopy (TEM) (Tecnai F20, Fei, Hillsborough, OR, USA) was used to observe the interior structure of materials. The thermal stability of the materials was evaluated by a thermal gravimetric analyzer (TGA) (TG/DTA7300, Seiko, Japan). A Brunauer–Emmett–Teller (BET) (ASAP 2460, Micromeritics, Atlanta, Georgia, USA) method was used to evaluate the specific surface area and pore size of the materials. An energy dispersive X-ray (EDX) with a JXA-8230 (Jeol, Japan) provided a chemical components analysis of the samples to determine their chemical composition when utilized it in combination with scanning electron microscopy (SEM) (S—3700N, Hitachi, Japan).

### 2.3. Materials Synthesis

MCM-41 synthesis referred to by Song et al. in [30]. Briefly, CTAB (1.25 g) was dissolved in 490 mL 14 wt% NH_4_OH and stirred for 5 min. Then, 10 mL of TEOS was added slowly into the solution and stirred at room temperature for 2 h. The precipitation was filtered, dried, and finally calcined at 550 °C for 6 h in the air to remove templates.

Surface grafting techniques were used to modify the TTIP on MCM-41 and the subsequent material was noted as MCM-T. One gram of MCM-41, 20 mL of anhydrous toluene and 50 µL of TTIP were added in a three-neck flask [31]. After sealing with two glass stoppers and a condenser pipe, the mixture was refluxed with vigorous stirring under a heating jacket for 2 h at 110 °C. The MCM-T was washed with anhydrous toluene and water. The material was dried in an oven at 80 °C for 24 h.

MCM-T functionalization with SDS was referred to by Bing et al. in [32]. Functionalized MCM-T was named as MCM-T-H. The synthetic procedure of MCM-T-H was as follows: 5 g of SDS was dissolved in 195 mL of deionized water under magnetic stirring until the SDS was dissolved completely. Then, 1 g of MCM-T was added, and the slurry was stirred continuously at room temperature for 1 h to obtain a good dispersion. The filtered MCM-T-H was then washed with ethanol and dried at 80 °C overnight.

### 2.4. Fabrication of MCM-T-H Coatings

As shown in Figure 1, 100 mg of PAN was mixed with 2 mL of DMF at 90 °C for 30 min to form a viscous binder solution, and then cooled to room temperature. Then, 20 mg of MCM-T-H was added into the binder solution and stirred for 12 h. The bare SPME Arrow was cleaned with ethanol and water to remove impurities and oil stains. Then, after etching with concentrated HCl to form a rough surface, it was washed with deionized water and dried at room temperature. After that, the bare SPME Arrow was gently immersed in the MCM-T-H/PAN mixture for five cycles. The length of the coated area on the metal bar was 1 cm, and the excess coating was scraped off with a knife. The MCM-T-H coated SPME Arrow (MCM-T-H-SPME Arrow) was aged in the GC inlet at 250 °C overnight.

### 2.5. SPME Arrow Procedures

First, 10 mL of the working solutions (or real sample solutions) was added into a 20 mL headspace vial and mixed with 10 µL of derivatization reagent IBCF. Then, the sample vial was sealed with a PTFE/silicone septum screwcap and placed on the magnetic stirrer for stirring. The direct immersion technique was used for the sampling with the SPME Arrow. The MCM-T-H-SPME Arrow punctured the septum and the coating part was exposed in the liquid sample for derivatized BAs extraction. After that, the SPME Arrow was inserted into the GC inlet for thermal desorption and GC–MS analysis. The desorption temperature was 250 °C and the desorption time was 5 min. The oven was programmed according to the following temperature program: the temperature was increased from 50 to 100 °C at 50 °C min^−1^, held at 100 °C for 1.2 min, increased to 160 °C at 10 °C min^−1^, and finally ramped to 280 °C at 25 °C min^−1^, and held for 12 min [14]. The total running time of the program was 25 min. Helium was used as the carrier gas and the gas flow rate was 1.0 mL min^−1^. The temperature of the transfer line, ion source, and analyzer were 280, 230, and 150 °C, respectively. Derivatized BAs were ionized using the electron ionization (EI) mode and scanned using the full scanning mode.

### 2.6. Real Sample Analysis

Pork and mackerel were used as the template food samples and they were purchased from local supermarkets in Ningbo. Pork and mackerel were sampled at the same time over seven consecutive days. First, 10 g of the sample was placed in a centrifugal tube, followed by adding 20 mL of 5% TCA, and the mixture was homogenized for 2 min. The supernatant was collected after centrifugation. The above operations were repeated twice then the supernatants were merged into a 50 mL volumetric flask and the final volume was fixed to 50 mL using 5% TCA. Finally, the pretreated samples were stored at 4 °C. The pH of the working solution was adjusted to 11 by using 2 mol L^–1^ NaOH. For the SPME Arrow extraction, 10 mL of the pretreated real sample was added into a 20 mL headspace vial and mixed with 10 µL of derivatization reagent IBCF for the subsequent SPME procedures.

## 3. Results and Discussion

All materials (MCM-41, MCM-T, and MCM-T-H) were characterized by FT-IR, XRD, BET and SEM (Section 3.1). The optimal synthesis conditions of MCM-T-H were determined by adjusting the amounts for the addition of CTAB, TTIP and SDS (Section 3.2). The optimal coating was determined by exploring the amount of MCM-T-H in the dipping mixture and the coating cycle (Section 3.3). After that, SPME procedures include sample volume, derivatization reagent amount, agitation speed, extraction and desorption steps were optimized, and the validated method was applied to real samples (Section 3.4, Section 3.5 and Section 3.6).

### 3.1. Characterization

**Figure 2:**
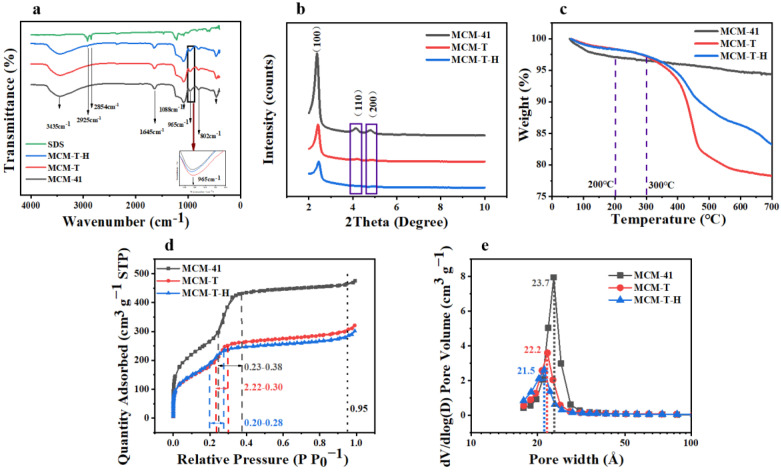
(**a**) FTIR spectra of MCM−41, MCM−T, MCM−T−H, and SDS; (**b**) XRD spectra of MCM−41, MCM−T, and MCM−T−H; (**c**) TGA patterns of MCM−41, MCM−T, and MCM−T−H; (**d**) nitrogen adsorption–desorption isotherms of MCM−41, MCM−T, and MCM−T−H; and (**e**) pore size distribution of MCM−41, MCM−T, and MCM−T−H.

Figure 2a shows the FTIR spectrum of MCM-41, MCM-H, MCM-T-H, and SDS. In the spectrum of MCM-41, MCM-T, and MCM-T-H, the absorption peaks at 3435 cm^−1^ were assigned to the stretching and bending vibration modes of the hydroxyl groups of physically adsorbed water molecules [33]. The observed peak at 1645 cm^−1^ could be ascribed to the presence of a Si-OH group, which was the main characteristic group of MCM-41, which also existed in MCM-T and MCM-T-H. Two peaks at 1088 cm^–1^ and 802 cm^–1^ were ascribed to asymmetric and symmetric stretching vibrations of Si-O-Si [34]. The FTIR spectra of all three materials had a band at 965 cm^−1^, which was usually attributed to the presence of the Si-O stretching vibration [35]. However, some literature has shown that the band at 965 cm^−1^ was due to stretching vibration caused by the bonding of SiO_2_ tetrahedron with the Ti atom through the Ti-O-Si bond [36]. The increasing intensity with the Ti content can be used as the evidence that Ti was successfully grafted to the framework. On the other hand, the energy band of MCM-41 at 965 cm^–1^ is slightly smaller than that of MCM-T and MCM-T-H, which indicated that TTIP had been successfully modified on the surface of MCM-41. Both SDS and MCM-T-H had absorption peaks at 2923 and 2852 cm^–1^, which were the stretching vibration peak of -CH_2_ [37]. They did not appear in MCM-41 and MCM-T, indicating that SDS had been successfully grafted onto the silicon surface.

Typical small-angle XRD patterns of three materials were shown in Figure 2b. MCM-41, MCM-T, and MCM-T-H had strong sharp diffraction peaks on the (1 0 0), (1 1 0), and (2 0 0) reflection surfaces, which usually represent the ordered hexagonal mesoporous structure [38]. These diffraction peaks indicated that MCM-T and MCM-T-H still share the same hexagonal structure as MCM-41 after Ti and SDS modification. With the addition of Ti and SDS, the diffraction intensity decreased significantly, which was due to the slight deformation of some pore channels and wall pores caused by the incorporation of Ti and SDS in MCM-41 [35]. 

Three materials show a type IV adsorption isotherm, indicating the mesoporous nature of the materials (Figure 2d). The adsorption isotherms of MCM-41 and functionalized materials have a very narrow H4 type loop, and the adsorption and desorption curves were reversible and almost coincident, which was related to the existence of slit holes in mesoporous materials [39]. The presence of pronounced and strong adsorption peaks between 0.23–0.38, 0.22–0.30, and 0.20–0.28 nm (Figure 2e) is from MCM-41, MCM-T, and MCM-T-H, respectively, which were due to the filling of uniform pores of the hexagonal lattice [40]. These results indicated the presence of hexagonal cylindrical channels, a key feature of the MCM-41. 

**Figure 3:**
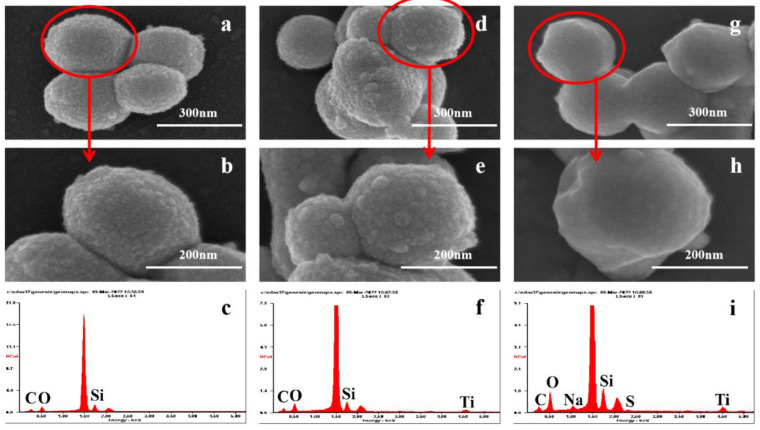
SEM images of (**a**) MCM−41; (**b**) MCM−41; (**d**) MCM−T; (**e**) MCM−T; (**g**) MCM−T−H; and (**h**) MCM−T−H. EDS spectra of (**c**) MCM−41; (**f**) MCM−T; and (**i**) MCM−T−H.

In order to further study the surface morphology characteristics of the three materials, SEM and EDS characterizations were carried out (Figure 3). The particle size of MCM-41 was about 200–350 nm (Figure 3a,b) with a rough surface and hexahedral structure. After functionalization with TTIP, Ti was attached to the surface of MCM-41 (Figure 3f), forming a coarser surface morphology (Figure 3d,e). Figure 3g–i shows that SDS, which contained S and Na, successfully grafted onto the material surface and made the surface of the morphology smoother. Functionalization did not obviously change the morphology of the hexahedron. This was consistent with the results of TEM (Figure S2), and Figure S2b,d shows that uniform pores were distributed in the material.

### 3.2. Optimization of the Coating Materials

**Figure 4:**
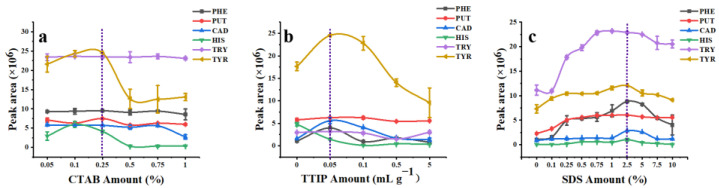
Optimization of MCM−T−H synthesis, (**a**) the amount of CTAB; (**b**) the amount of TTIP; (**c**) the amount of SDS.

MCM-41 is a kind of amorphous mesoporous silica material with a unique hexahedral structure [41]. Mesopore size can be adjusted by altering the amount of CTAB. Overall, the addition amount of CTAB had little effect on the extraction efficiency of MCM-41 for all target analytes except TYR (Figure 4a) (TTIP and SDS amounts were 0% in these experiments). When the additional amount of CTAB was 0.25%, MCM-41 had the best adsorption performance for PHE, PUT, TRY, and TYR. Under this condition, MCM-41 with a higher specific surface, larger pore volume, and appropriate mesoporous pore size (Table S1, surface area: 893.33 m^2^ g^–1^; pore diameter: 2.37 nm; total pore volume: 0.73 cm^3^ g^–1^) showed the highest extraction capacity to derivatized BAs. Therefore, 0.25% of CTAB was selected as the optimal additive amount to synthesize MCM-41.

Incorporation of Ti into mesoporous silica generates a large number of Lewis and Brønsted acid sites, which further improves the acidity of the material and thus enhances the adsorption affinity to BAs [20]. MCM-T showed good extraction efficiency for five kinds of derivatized BAs except HIS when the addition amount of TTIP was 50 μL g^–1^ (Figure 4b). Compared to MCM-41, MCM-T showed better extraction efficiency to derivatized BAs, which indicated the chemical affinity enhancement of MCM-T to BAs after Ti modification. As we expected, surface grafting decreased the pore sizes and pore volumes of MCM-41 (pore diameter, pore volume, and specific surface area of MCM-T were 2.22 nm, 0.50 cm^3^ g^–1^, and 587.44 m^2^ g^–1^, respectively (Table S1)). Pore size and pore volume reduction was because of the modification of terminal groups by TTIP that were bolted in the inner pores of MCM-41. Surface area reduction of MCM-T (587.44m^2^ g^–1^) was partly due to the structural collapse of MCM-41 upon incorporation with denser Ti species [42].

In this study, the MCM-T series material coated SPME Arrow was used for the extraction of derivatized BAs from an aqueous solution, therefore the existence of water molecules was an important factor that could not be ignored. The hydrophobic surface of the adsorbent material could improve the compatibility between particles and the derivatized BAs and reduced the interference of water molecules. Therefore, MCM-T was further functionalized with SDS to obtain hydrophobic surfaces and thereby named as MCM-T-H. Extraction efficiency of MCM-T-H to derivatized BAs increased dramatically due to their hydrophobic surface and the best modification concentration of SDS was 2.5% (Figure 4c). The thickness of the modification layer was approximately 8.8 nm (Figure S2d). Texture properties of MCM-T-H was maintained (surface area: 604.52 m^2^ g^–1^; pore diameter: 1.87 nm; total pore volume: 0.47 cm^3^ g^–1^) (Table S1). Surface grafting with SDS further decreased the pore sizes and pore volumes of the materials but greatly improved the extraction efficiency of derivatized BAs which indicated the importance of modifying the MCM-41 substrate.

### 3.3. Optimization of the SPME Arrow Coating

#### 3.3.1. MCM-T-H Amount

**Figure 5:**
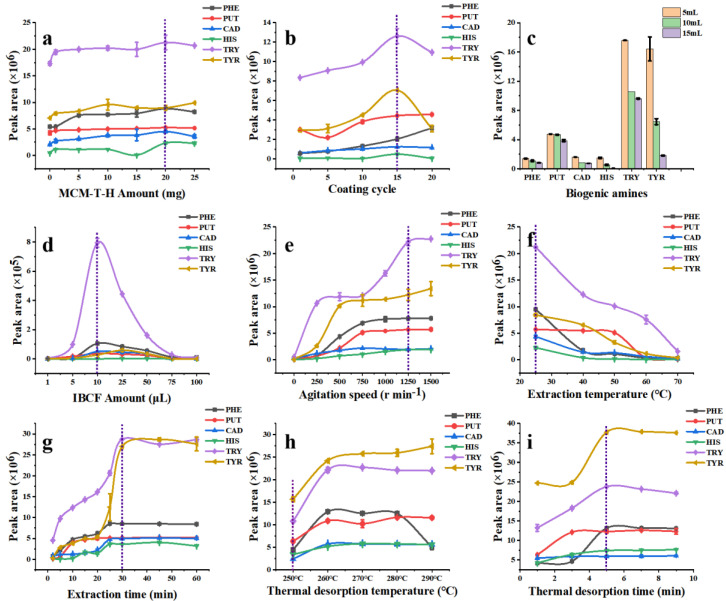
Optimization of SPME Arrow procedures, (**a**) MCM−T−H amount; (**b**) coating cycle; (**c**) sample volume; (**d**) derivatization reagent amount; (**e**) agitation speed; (**f**) extraction temperature; (**g**) extraction time; (**h**) thermal desorption temperature; (**i**) thermal desorption time.

Figure 5a shows the effect of the material amount on the extraction efficiency of the MCM-T-H coated SPME Arrow. The SPME Arrow showed the best adsorption performance for five derivatized BAs except TYR when the material addition amount was 20 mg which was due to the increased functional groups. However, a higher addition amount did not obviously increase the extraction efficiency which is probably because more adsorption sites hindered the diffusion path in MCM-T-H and therefore reduced the effective specific surface area. Therefore, the best addition amount of MCM-T-H was 20 mg in 2 mL of DMF.

#### 3.3.2. Coating Cycle

During the preparation of the SPME Arrow, the increase of coating cycles could raise up the thickness of the coating and therefore increase its extraction capacity. The bare SPME Arrow had an outer diameter of around 320 μm (Figure S3a). With an increase of coating cycles to 15 times (Figure S3b), the coating thickness was approximately 4 μm (Figure S3e) and it showed the highest extraction efficiency to derivatized BAs (Figure 5b). After 20 coating cycles, its extraction efficiency was not obviously increased. Moreover, the MCM-T-H coating was not damaged after 10 extraction and desorption cycles (Figure S3c), but it had partially fallen off after 50 extraction and desorption cycles (Figure S3d), which indicated its relatively high-physical stability. On the other hand, when the coating was too thick, it hindered the diffusion path and reduced the adsorption performance. Therefore, the coating cycle for subsequent experiments was 15 times.

The MCM-T-H-SPME Arrow was compared with the commercial DVB/CAR/PDMS-SPME Arrow, and the result is shown in Figure S4. In a preliminary study, the DVB/CAR/PDMS-SPME Arrow showed the best extraction capacity to derivatized BAs under optimal extraction and desorption conditions (data not shown) and that is why it was the only commercial coating to be compared. The extraction efficiency of the MCM-T-H-SPME Arrow was better than that of the commercial SPME Arrow for six derivatized BAs because MCM-T-H has better affinity to derivatized BAs and the hydrophobic surface significantly reduced the interference of water molecules.

### 3.4. Optimization of SPME Procedures

#### 3.4.1. Sample Volume

Firstly, the effect of sample volume to extraction efficiency of MCM-T-H coating was studied. Theoretically, a smaller sample volume leads to higher extraction efficiency because the turbulence effect is stronger in a smaller sample volume under the same stirring speed and thus the diffusion rate of analytes increases in the boundary layer around the coating. A mixture containing six kinds of BAs (1 mg L^−1^) was used. As predicted, the MCM-T-H coating showed the best extraction efficiency when the sample volume was 5 mL (Figure 5c), but it easily caused damage to the coating due to the collision between the SPME Arrow tip and the magnetic stirrer. Therefore, 10 mL of sample was used in further studies after considering the practical situation.

#### 3.4.2. Derivatization Reagent Amount

IBCF was selected as a derivatization reagent because alkyl groups in IBCF can replace H^+^ in BAs and complete the conversion reaction of alkyl chloroformate (Figure S5) [43]. IBCF is an acylation reagent, which can decrease the polarity of amines and aid in decreasing nonspecific adsorption effects [44]. In addition, the reaction can be carried out in an alkaline aqueous solution, which greatly simplifies the sample pretreatment process. The fragment, retention time, and structure formula for procedure of determination of BAs based by application of GC–MS technique is shown in Table S2. The derivatization products, which have lower polarity and lower boiling [43], can be detected by GC–MS. The addition amount of IBCF was therefore studied because it is critical in a derivatization reaction. The sample pH was adjusted to 11 by NaOH (2 mol L^−1^) since a BAs–IBCF reaction was easier to carry out in a basic solution. IBCF is acidic, therefore, an excess of IBCF significantly reduced the pH, which hindered the derivative reaction process. However, too little IBCF could not guarantee the completion of the derivatization reaction. After a compromising consideration and according to the optimization results (Figure 5d), 10 μL of IBCF was added into a 10 mL sample solution.

#### 3.4.3. Agitation Speed

With the increase of the stirring speed, the extraction efficiency of the SPME Arrow to derivatized BAs analytes was also improved (Figure 5e). This phenomenon can be explained as when the stirring speed was increased, the turbulence effect was promoted and the diffusion rate of the analytes to the boundary layer surrounding the coating was higher, which resulted in a higher extraction efficiency. When the stirring speed was increased to 1250 r min^–1^, the extraction efficiency of five derivatized BAs was saturated except TYR. However, when the stirring speed was 1500 r min^–1^, the coating could not be fully immersed under the sample solution due to the vortex phenomenon, which could cause large experimental errors. Therefore, the optimal stirring speed was 1250 r min^–1^ and was used in subsequent experiments.

#### 3.4.4. Extraction Temperature and Time

Figure 5f shows the effect of adsorption temperature to MCM-T-H extraction efficiency. With the temperature increasing, the extraction efficiency decreased which indicated that the adsorption process was exothermic, and desorption may occur at higher temperature due to more severe thermal movement of target analytes, which could lead to the reduction of extraction efficiency. Therefore, the optimum adsorption temperature was set to 25 °C. 

Extraction time is an important parameter that significantly affects the adsorption performance because the extraction mechanism of SPME technique. It can be seen in Figure 5g that extraction equilibrium was reached when extraction time was 30 min and prolonged extraction time did not improve the extraction efficiency obviously. This phenomenon can be interpreted as free active sites of MCM-T-H that were occupied by derivatized BAs after 30 min, so the adsorption equilibrium was reached. Therefore, the optimum extraction time was set to 30 min.

#### 3.4.5. Thermal Desorption Temperature and Time

Desorption is an indispensable procedure in SPME, and thermal desorption is available to desorb derivatized BAs directly in the GC inlet, therefore desorption temperature and time were optimized. According to the experimental results (Figure 5h), 260 °C was the appropriate temperature to desorb most analytes into the GC. However, MCM-T-H was not thermally stable over 250 °C due to the grafted SDS groups (Figure 2c). Therefore, 250 °C was selected as the optimal thermal desorption temperature. Thermal desorption time was further optimized in the range of 1–9 min. Compared with static desorption, thermal desorption is faster and more comprehensive. Results (Figure 5i) showed that 5 min is enough to desorb analytes completely.

### 3.5. Linearity, Detection Limit, and Precision

A SPME Arrow was combined with GC–MS to realize the BAs analysis. The linearity of the MCM-T-H-SPME Arrow GC–MS method was therefore studied. The results showed that linear correlation coefficients of PHE, PUT, CAD, HIS, TRY, and TYR were 0.9969, 1, 0.9993, 0.9956, 0.9998, and 0.9944, respectively (Table 1). The LOD and LOQ were calculated from S/N of three and ten, respectively, were in the range of 1.1–26.8 μg L^–1^ and 3.5–89.3 μg L^–1^, respectively. Repeatability was verified by intraday and interday analyses results. The RSD of intraday and interday were 0.6–6.7% and 1.6–9.8%, respectively. Compared to reported methods in the literature (Table 2), this work showed relatively lower LODs and LOQs, which indicated the high sensitivity of this method. In addition, our developed method also showed satisfactory repeatability and exhibited good applicability for derivatized BAs analysis in different food samples. Merits of the developed BAs analytical method indicated its feasibility for food sample analysis. 

### 3.6. Pork and Mackerel Samples Analyses

The applicability of our developed method was validated by monitoring BA concentrations in pork and mackerel, which were stored at room temperature (22 ± 2 °C) for seven days in a row. Over those days, variations of BAs in pork and mackerel from each day were analyzed with triplicate measurements. In addition, pork and mackerel samples on the third and sixth storage days were used for a recovery test to evaluate the precision and accuracy of this method. Monitoring results are summarized in Figures S6 and S7 and recovery results are listed in Table 3 and Table 4. The GC chromatograms and mass spectrums of BAs in pork and mackerel are shown in Figure S8. The concentrations of PHE, PUT, CAD, and TYR in pork in seven days ranged from 0.12–0.77 mg kg^–1^ (PHE), 0.9–5.12 mg kg^–1^ (PUT), 0.10–9.48 mg kg^–1^ (CAD), to 0.43–3.25 mg kg^–1^ (TYR), respectively. The concentrations of PHE, PUT, CAD, HIS, TRY, and TYR in mackerel were varied in the range of 5.2–234 mg kg^–1^ (PHE), 4.5–457 mg kg^–1^ (PUT), 0.5–585 mg kg^–1^ (CAD), 6.8–657 mg kg^–1^ (HIS), 0.6–46.6 mg kg^–1^ (TRY), and 0.3–696 mg kg^–1^ (TYR), respectively. After three days of storage, the mackerel has spoiled and was inedible while the BAs were still at a quite low concentration in pork which is probably due to the higher protein, water, and bacterial contents in mackerel. BA recoveries from pork and mackerel were 78.5–123% and 74.6–118%, respectively. Obviously, some recovery results were higher than 100% which could be for two reasons. First, the operation error and instrumental error leads to deviation of recoveries. Second, the homogeneity of the sample is also an important factor which affects the recovery result. The spiked levels of several BAs were higher than the LOQ values because we spiked the BAs based on their concentrations in the pork and fish samples. For example, the CAD concentration in the mackerel on day three was 352 ± 22 mg kg^–1^, therefore 350 mg kg^–1^ of CAD was spiked into the fish sample to evaluate the recovery of CAD using our developed method. On the other hand, since some BA concentrations exceeded the linear range of our method, we processed a preliminary experiment to estimate the BAs’ concentration and then we diluted the extraction solutions of pork and fish samples using a basic solution (pH = 11). Overall, those results indicated that the MCM-T-H coating was stable and reliable to enrich derivatized BAs efficiently from complicated food matrices. In addition, the developed MCM-T-H-SPME Arrow GC–MS method is applicable to precisely monitor BAs variation with satisfactory recovery.

Overall, a SPME Arrow GC–MS method based on the hydrophobic mesoporous silica was successfully developed for qualitative and quantitative analysis of six BAs in pork and fish. The mesoporous MCM-41 was grafted with titanium, and thereby a large number of Lewis and Brønsted acid sites were generated, which further improved its acidity, and the extraction efficiency of the acidified MCM-41 to derivatized BAs was obviously increased. SDS was further modified on MCM-T to increase its hydrophobicity so that the interference of water molecules could be avoided as much as possible. According to the results, the hydrophobic MCM-T-H coating has a high-extraction affinity to derivatized BAs rather than water molecules. The MCM-41 series materials were comprehensively characterized by multiple characterization techniques and the selective extraction mechanism of MCM-T-H to derivatized BAs was studied. The SPME Arrow GC–MS method developed showed excellent sensitivity, selectivity, and reproducibility and it also showed good applicability for the detection of six BAs from pork and fish samples.

## 4. Conclusions

To summarize, hydrophobic MCM-T-H was prepared and used as the coating material of an SPME Arrow. The average thickness of the MCM-T-H coating was 4 µm with RSD less than 9.8% (*n* = 3), and the coating was stable up to 50 extraction and desorption cycles. Compared to the commercial SPME fiber, the MCM-T-H-SPME Arrow has the advantages of being low cost and robust. In addition, the MCM-T-H-SPME Arrow has a better extraction performance for the six derivatized BAs than the commercial DVB/CAR/PDMS-SPME Arrow. The MCM-T-H-SPME Arrow GC–MS method developed here can accurately monitor the variation of BAs in pork and fish samples with good recovery. Therefore, this method provides a promising alternative to conventional BA detection methods and extends the application of the SPME Arrow to food safety. Moreover, the material is simple to synthesize with a low cost, which will greatly reduce labor and other costs in the traditional food monitoring field.

## Figures and Tables

**Table 1:** The standard curves, linear range, LOD, LOQ, and repeatability of the SPME Arrow GC–MS method for the determination of six biogenic amines.

Analytes	Standard Curves	Linearity Range (μg L^−1^)	R^2^	LOD (μg L^−1^)	LOQ (μg L^−1^)	Repeatability (RSD, %)	
						Intraday (*n* = 3)	Interday (*n* = 3)
PHE	y = 2940x − 53049	10–1000	0.9969	2.4	7.3	6.7	2.8
PUT	y = 184x − 13167	100–1000	1	18	60	0.6	1.6
CAD	y = 478x + 1741	10–1000	0.9933	2.0	6.7	0.6	1.6
HIS	y = 391x − 2108	100–1000	0.9956	27	89	5.5	9.8
TRY	y = 2907x − 22115	10–1000	0.9998	2.4	8.1	5.6	4.6
TYR	y = 2356x + 21113	10–1000	0.9944	1.1	3.5	2.8	2.8

**Table 2:** Comparison of reported methods and the method developed in this study.

Methods	LODs (μg L^−1^)	LOQs (μg L^−1^)	Linearity Range (μg L^−1^)	Analytes	Samples	RSD (%)	References
^a^ D-SPME-HPLC	9–17	28–49	50–150,000	TYR, PUT, CAD, HIS	Smoked fish	0.53–5.16	[45]
^b^ SALLE-HPLC- FLD	7.5–1600	23–4900	1000–25,000	MET, ETH, DIM, PHE, ISO, PUT, CAD, HIS, TYR, SPD, SPM	Fish and meat	1.7–10	[46]
^c^ HPLC-FLD	20–100	60–300	100–10,000	PUT, CAD, SPD, SPM, HIS	Fish	7.4–14	[47]
SPME-GC–MS	1.1–27	3.5–89	10–1000	PHE, PUT, CAD, HIS, TRY, TYR	Fish and Pork	0.6–9.8	This work

^a^ Dispersed solid-phase microextraction high-performance liquid chromatography; ^b^ salting-out assisted liquid–liquid extraction high-performance liquid chromatography fluorescence detector; ^c^ high-performance liquid chromatography fluorescence detector.

**Table 3:** Recovery of biogenic amines in pork on day three and six.

Analytes	Day Three			Day Six		
	Concentration (mg kg^–1^)	Spiked Level (mg kg^–1^)	Recovery (%)	Concentration (mg kg^–1^)	Spiked Level (mg kg^–1^)	Recovery (%)
PHE	0.19 ± 0.01	0.18	88.3	0.77 ± 0.01	0.75	122.8
PUT	4.19 ± 0.06	4.0	94.6	5.12 ± 0.09	5.00	111
CAD	2.45 ± 0.96	2.5	111.8	9.48 ± 3.79	9.50	85.4
HIS	ND			ND		
TRY	ND			ND		
TYR	3.25 ± 0.01	3.25	78.5	1.04 ± 0.39	1.00	99.7

**Table 4:** Recovery of biogenic amines in mackerel on day three and six.

Analytes	Day Three			Day Six		
	Concentration (mg kg^–1^)	Spiked Level (mg kg^–1^)	Recovery (%)	Concentration (mg kg^–1^)	Spiked Level (mg kg^–1^)	Recovery (%)
PHE	13.0 ± 1.8	15	101.2	168 ± 10	175	74.6
PUT	77.8 ± 11.0	80	105.4	451 ± 13	450	83.4
CAD	352 ± 22	350	118.3	406 ± 17	400	94.8
HIS	224 ± 28	220	83.6	136 ± 27	138	81.5
TRY	8.2 ± 1.5	7.5	109.7	42 ± 2	37.5	86.4
TYR	56.6 ± 17.0	57.5	109.2	250 ± 15	250	102.2

## Data Availability

Data is contained within the article or Supplementary Materials.

## Supplementary Materials

The following supporting information can be downloaded at: https://www.mdpi.com/article/10.3390/foods12030578/s1, Figure S1: Synthesis route of MCM-T-H; Figure S2: TEM images of (**a**) MCM–41; (**b**) MCM–41; (**c**) MCM–T–H; and (**d**) MCM–T–H; Figure S3: (**a**) Bare SPME Arrow; (**b**) SPME Arrow coated with MCM–T–H; (**c**) MCM–T–H–SPME Arrow used 10 times; (**d**) MCM–T–H–SPME Arrow used 50 times; and (**e**) coating thickness; Figure S4: Comparison of extraction efficiency between MCM–T–H and DVB/CAR/PDMS-coated SPME Arrows; Figure S5: Schematic representation of the derivatization of BAs with isobutyl chloroformate; Figure S6: Variations of the biogenic amines content in pork over 7 days; Figure S7: Variations of the biogenic amines content in mackerel over 7 days; Figure S8: GC chromatograms (**A**) and mass spectrums (**B**) of pork and mackerel; Table S1: Textural properties of MCM–41 series materials; Table S2: Fragments, retention times, and structural formulas for the procedure of determination of BAs based on the application of the GC–MS technique.
