# Supplementary material for: Hydrophobic Mesoporous Silica-Coated Solid-Phase Microextraction Arrow System for the Determination of Six Biogenic Amines in Pork and Fish
Source: Foods. 2023 Jan 28;12(3):578. doi: 10.3390/foods12030578 (PMC9914681; doi:10.3390/foods12030578)
Supplement: Supplementary file 1 [file foods-12-00578-s001.zip › foods-2151223-supplementary.pdf]

foods

Article

Electric Supplementary Material

Hydrophobic Mesoporous Silica-Coated Solid Phase Microextraction Arrow System for the Determination of Six Biogenic Amines in Pork and Fish

Mengfei Chen, Hangzhen Lan *, Daodong Pan and Tao Zhang

State Key Laboratory for Quality and Safety of Agro-products, Key Laboratory of Animal Protein Deep Processing Technology of Zhejiang Province, Zhejiang-Malaysia Joint Research Laboratory for Agricultural Product Processing and Nutrition and College of Food and Pharmaceutical Sciences, Ningbo University, Ningbo 315800, China

* Correspondence: lanhangzhen@nbu.edu.cn

Citation: Chen, M.; Lan, H.; Pan, D.; Zhang, T. Hydrophobic Mesoporous Silica-Coated Solid-Phase Microextraction Arrow System for the Determination of Six Biogenic Amines in Pork and Fish. *Foods* **2023**, *12*, 578. https://doi.org/10.3390/foods12030578

Academic Editors: Marco Beccaria and Chiara De Luca

Received: 24 December 2022
Revised: 18 January 2023
Accepted: 20 January 2023
Published: 28 January 2023

Copyright: © 2023 by the authors. Licensee MDPI, Basel, Switzerland. This article is an open access article distributed under the terms and conditions of the Creative Commons Attribution (CC BY) license (https://creativecommons.org/licenses/by/4.0/).

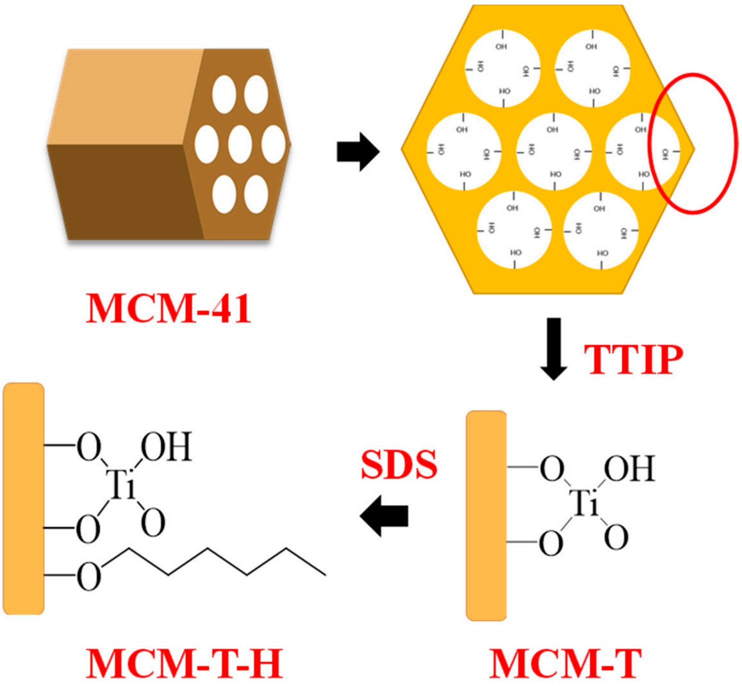

Figure S1. Synthesis route of MCM-T-H.

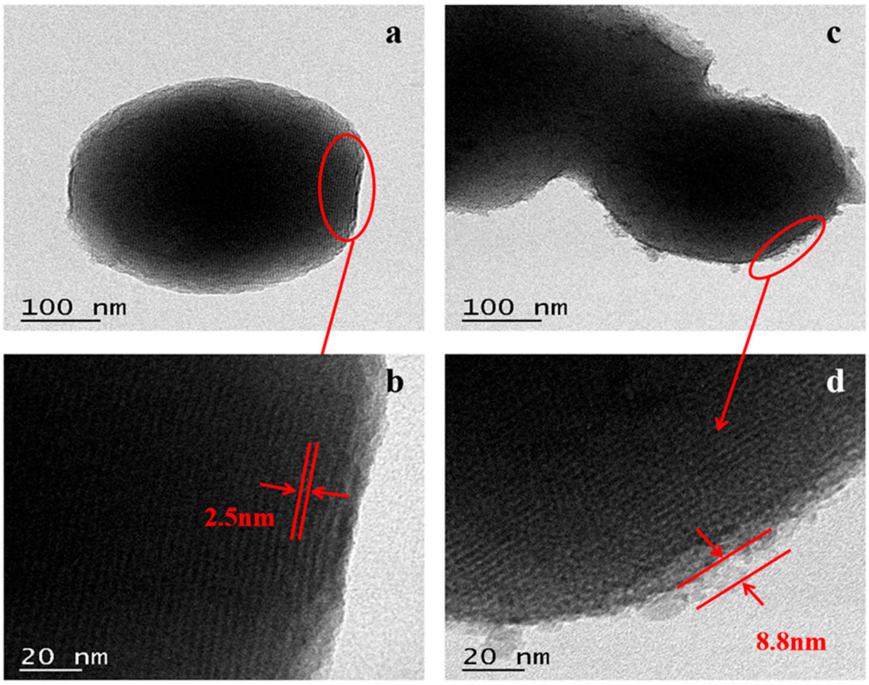

Figure S2. TEM images of (**a**) MCM–41; (**b**) MCM–41; (**c**) MCM–T–H; and (**d**) MCM–T–H.

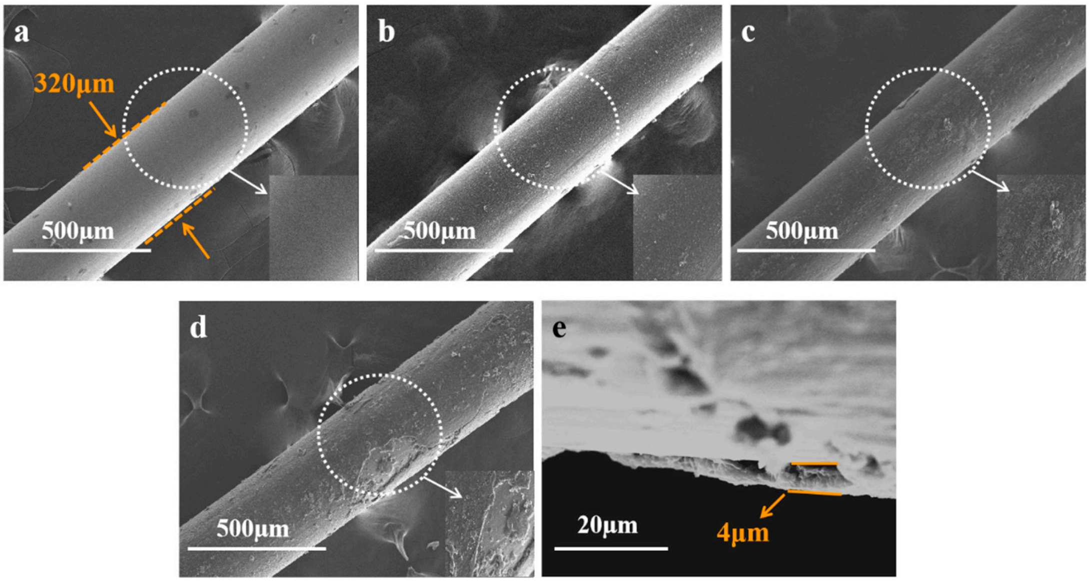

Figure S3. (**a**) Bare SPME Arrow; (**b**) SPME Arrow coated with MCM–T–H; (**c**) MCM–T–H–SPME Arrow used 10 times; (**d**) MCM–T–H–SPME Arrow used 50 times; and (**e**) coating thickness.

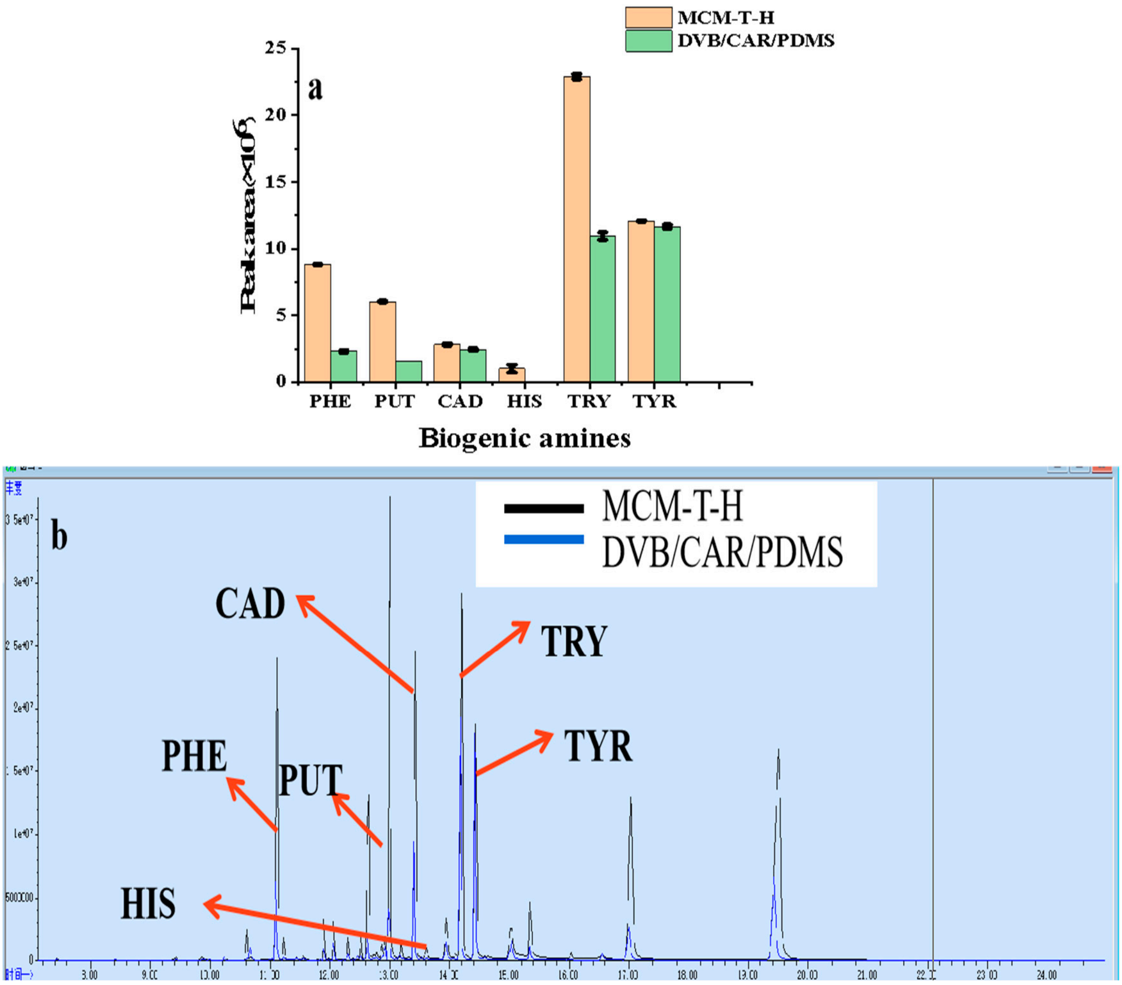

Figure S4. Comparison of extraction efficiency between MCM–T–H and DVB/CAR/PDMS-coated SPME Arrows.

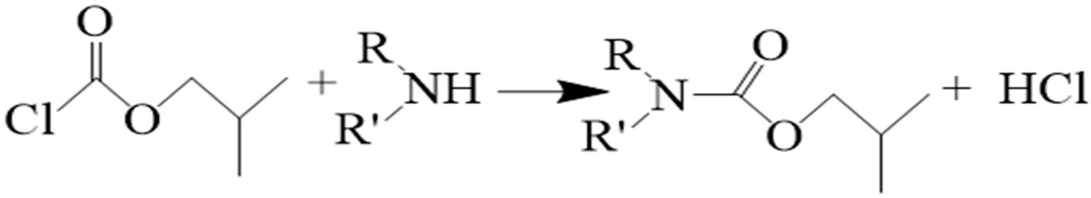

Figure S5. Schematic representation of the derivatization of BAs with isobutyl chloroformate.

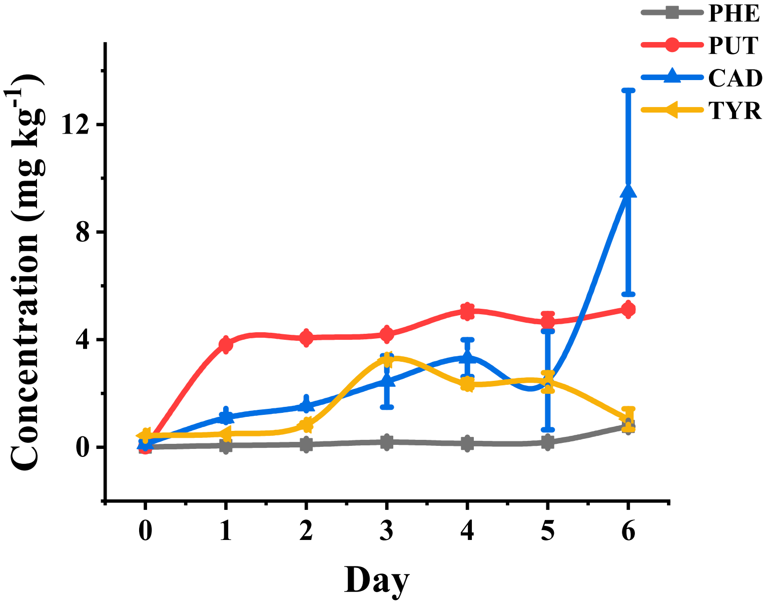

Figure S6. Variations of the biogenic amines content in pork over 7 days.

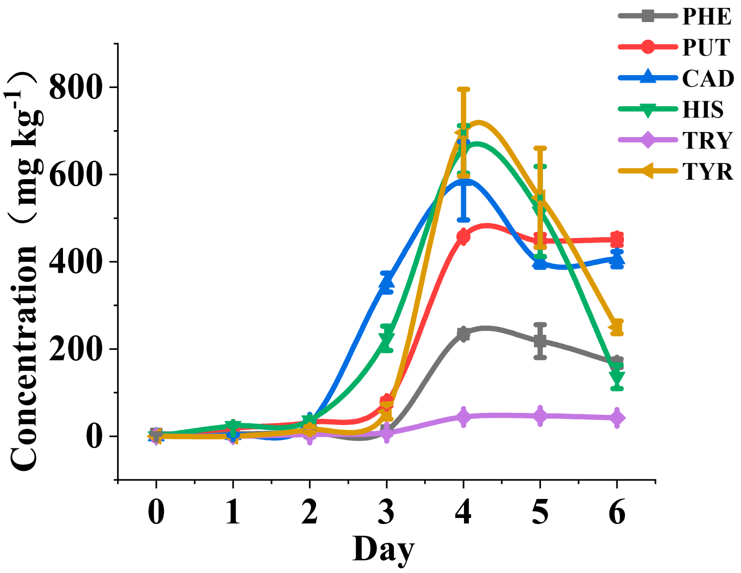

Figure S7. Variations of the biogenic amines content in mackerel over 7 days.

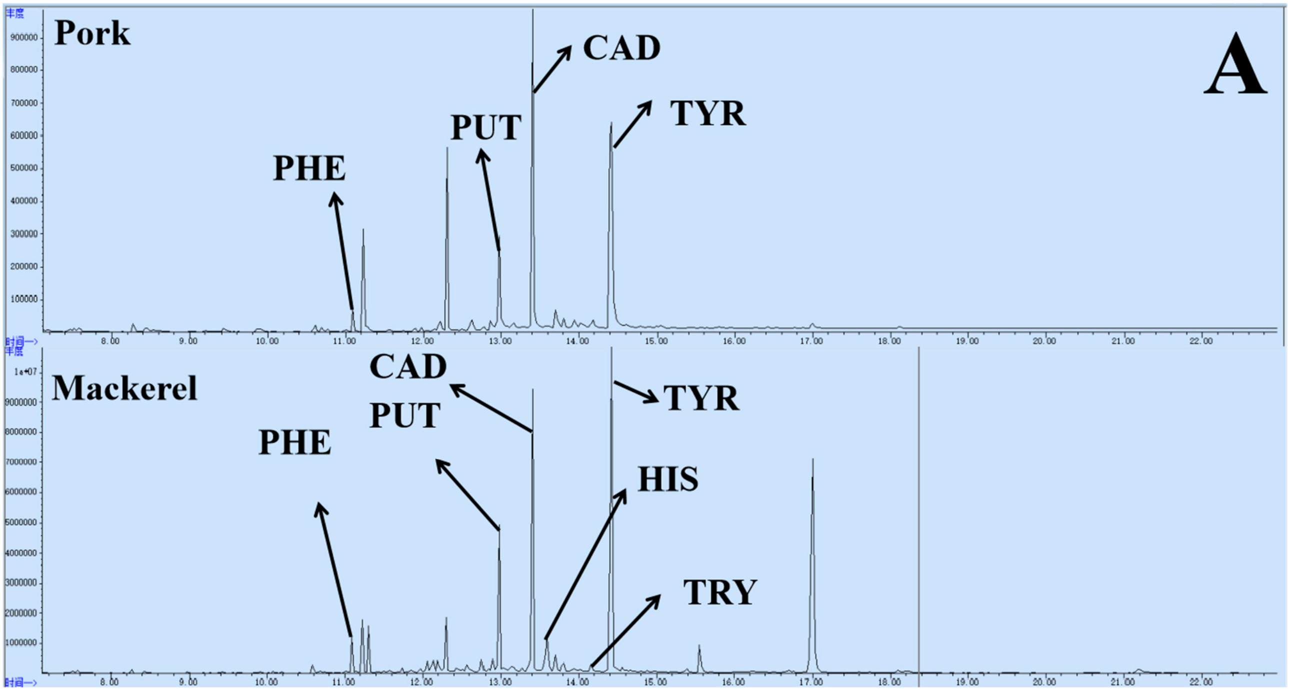

(**A**)

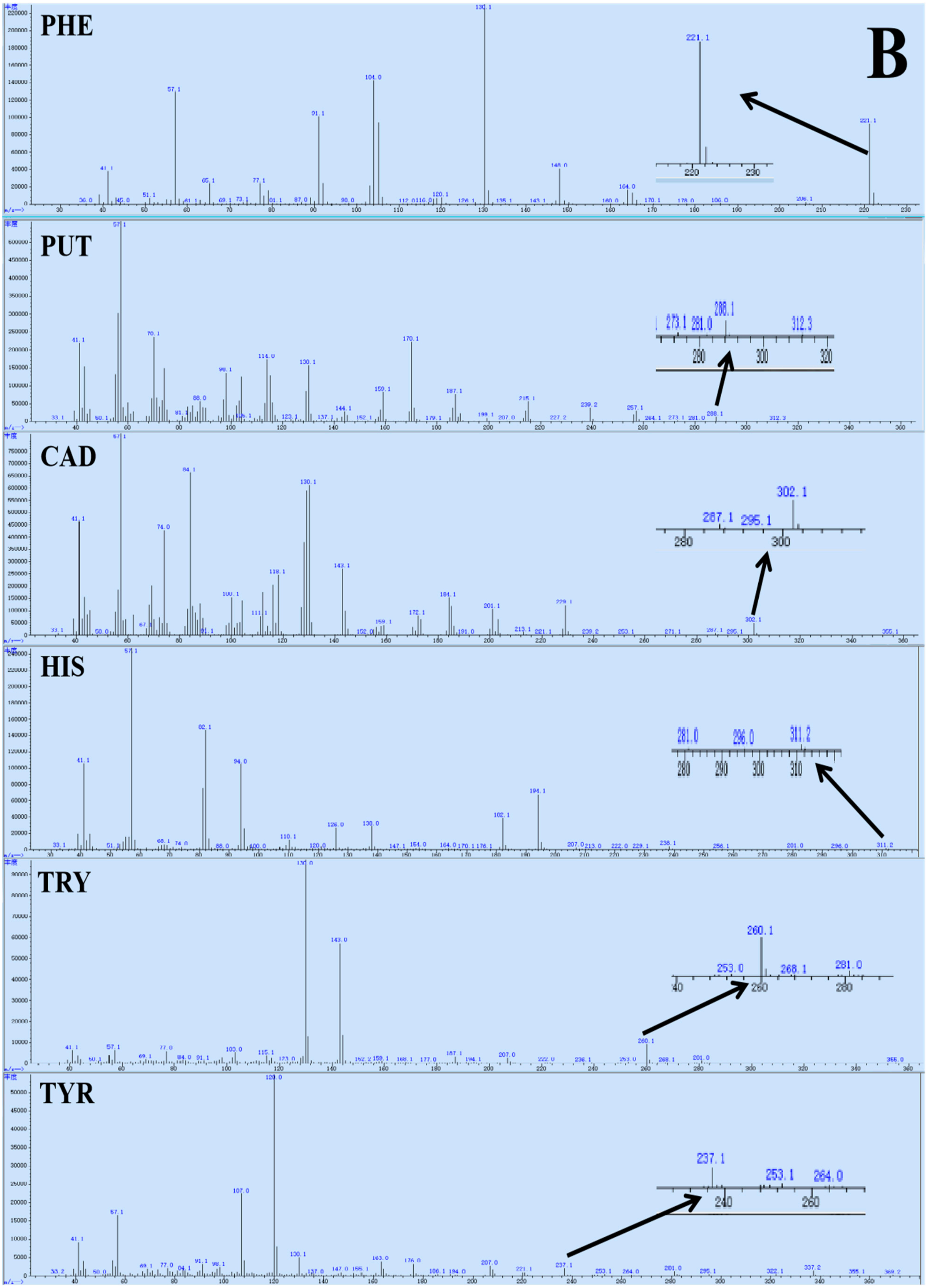

(**B**)

Figure S8. GC chromatograms (**A**) and mass spectrums (**B**) of pork and mackerel.

Table S1. Textural properties of MCM–41 series materials.

	BET Surface Area (m^2 g^{-1})	Pore Diameter (nm)	Total Pore Volume (cm^3 g^{-1})
MCM–41	893.3	2.37	0.73
MCM–T	587.4	2.22	0.50
MCM–T–H	604.5	1.87	0.47

Table S2. Fragments, retention times, and structural formulas for the procedure of determination of BAs based on the application of the GC–MS technique.

Analytes	*m*/*z*	Retention Time (min)	Chemical Structure	Derivatized Products
PHE	221	11.14	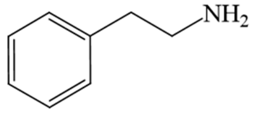	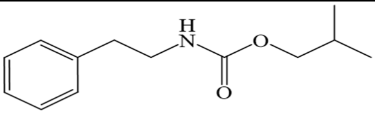
PUT	288	13.02	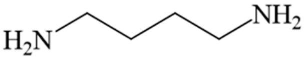	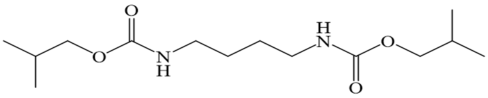
CAD	302	13.46	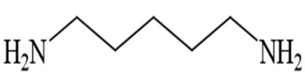	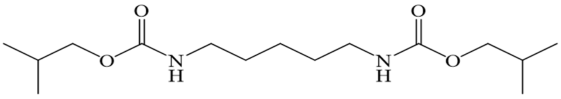
HIS	311	13.64	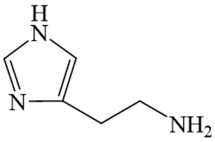	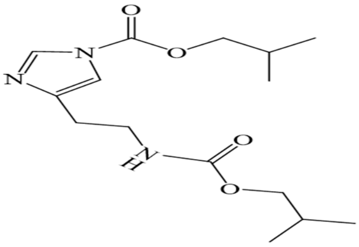
TRY	260	14.32	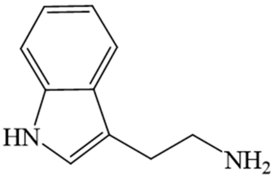	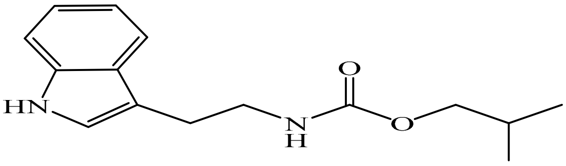
TYR	237	14.51	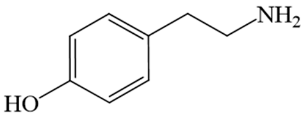	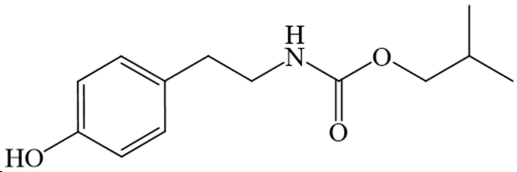
